# Diet impacts triple‐negative breast cancer growth, metastatic potential, chemotherapy responsiveness, and doxorubicin‐mediated cardiac dysfunction

**DOI:** 10.14814/phy2.15192

**Published:** 2022-04-19

**Authors:** Manuel U. Ramirez, Kenysha Y. J. Clear, Zipporah Cornelius, Alaa Bawaneh, Yismeilin R. Feliz‐Mosquea, Adam S. Wilson, Alistaire D. Ruggiero, Nildris Cruz‐Diaz, Lihong Shi, Bethany A. Kerr, David R. Soto‐Pantoja, Katherine L. Cook

**Affiliations:** ^1^ Department of Physiology and Pharmacology Wake Forest University Health Sciences Winston‐Salem North Carolina USA; ^2^ Department of Surgery‐Hypertension Wake Forest School of Medicine Winston‐Salem North Carolina USA; ^3^ 12279 Department of Pathology Wake Forest School of Medicine Winston‐Salem North Carolina USA; ^4^ Cardiovascular Sciences Wake Forest School of Medicine Winston‐Salem North Carolina USA; ^5^ Department of Cancer Biology Wake Forest School of Medicine Winston‐Salem North Carolina USA; ^6^ Comprehensive Cancer Center Wake Forest School of Medicine Winston‐Salem North Carolina USA

**Keywords:** cardiac damage, doxorubicin, drug resistance, fish oil, lung metastases, Mediterranean diet, triple‐negative breast cancer, Western diet

## Abstract

Anthracyclines are standard‐of‐care chemotherapy for the treatment of triple‐negative breast cancer (TNBC). However, high anthracyclines cumulative doses increase heart failure risk. Designing therapeutic strategies that ameliorate cardiac toxicities without compromising oncologic efficacy are important to improve TNBC outcomes and survivorship. The purpose of this study was to determine the impact of diet on TNBC chemotherapeutic responsiveness and development of chemotherapy‐induced cardiac damage. Female BALB/c mice fed a control, Western, Mediterranean, or Western + fish oil diet were injected with 1 × 10^6^ 4T1‐luciferase TNBC into the mammary fat pad. Tumors grew for 21 days before surgical tumor resection, then mice were treated with 3.3 mg/kg i.v. doxorubicin for 3 weeks. Vevo (R) cardiac ultrasound was performed. Female nu/nu mice were placed on diets before 1 × 10^5^ MDA‐MB‐231‐luciferase TNBC were injected via the tail vein to induce the development of lung metastases. Mice were treated with saline or 3.3 mg/kg i.v. doxorubicin for 3 weeks, and the development of metastases visualized by IVIS (R). Consumption of a high‐fat diet increased TNBC growth regardless of dietary pattern. Western diet‐fed mice developed lung metastases sooner and displayed increased lung metastatic lesion formation, which was not observed in Mediterranean diet‐fed mice. Western diet‐fed animals displayed worse cardiac function when compared with Mediterranean diet‐fed animals. Hearts from Western diet‐fed animals displayed increased fibrosis. Diet represents a modifiable component directly impacting tumor growth, antitumor chemotherapy efficacy, and cardiac toxicities. Our data suggest that the Mediterranean diet may reduce lung metastatic lesions formation and prevent the development of cardiac toxicities.

## Graphical abstract

Mediterranean diet consumption during chemotherapy reduces lung metastatic lesion formation while concurrently preventing the development of doxorubicin‐induced cardiac toxicities in female BALB/c mice when compared with Western diet‐consuming animals. Lung mets = lung metastases. Figure created using biorender.com.

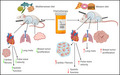

**Abbreviations**
ACT: Adriamycin, Cyclophosphamide, Taxol
DAB: 3,3′‐Diaminobenzidine
DOX: Doxorubicin
ER: Estrogen receptor α
EVOO: Extra virgin olive oil
IV: Intravenous
IVIS: In vivo imaging system
MRI: Magnetic resonance imaging
MUFA: Monounsaturated fatty acids
PREDIMED: Primary Prevention of Cardiovascular Disease with a Mediterranean Diet clinical trial
PUFA: Polyunsaturated fatty acids
TNBC: Triple‐negative breast cancer

## 1 INTRODUCTION

Breast cancer is the most frequently diagnosed cancer in women with approximately 281,000 new cases diagnosed each year (Siegel et al., 2021). Triple‐negative breast cancer (TNBC) represents around 10–15% of all breast cancer cases and predominately affects young and minority women (Foulkes et al., 2010). TNBC is highly aggressive and currently has no targeted therapeutic options, thereby limiting patients to chemotherapy (Perez et al., 2019). Several studies have demonstrated a strong link between obesity and increased risk of developing premenopausal TNBC (Pierobon & Frankenfeld, 2013; Sun et al., 2017). Furthermore, some studies reported a three‐fold higher mortality rate in obese women at diagnosis (Protani et al., 2010), indicating the important role obesity plays in modulating therapeutic responsiveness, promoting metastases, and/or the development of off‐target toxicities that limit treatment to decrease breast cancer survival (Guenancia et al., 2016).

In the United States, there are over 3.8 million breast cancer survivors, making the unique long‐term health concerns of this population a health priority. Breast cancer survivors face a greater burden of non‐cancer chronic diseases, such as cardiovascular disease, compared to their cancer‐free peers, leading to excess morbidity and reduced quality of life (Bradshaw et al., 2016; Gernaat et al., 2017; Patnaik et al., 2011, 2011). Anthracycline‐based strategies remain the standard chemotherapy for adjuvant and neoadjuvant treatment of TNBC (Isakoff, 2010). However, high cumulative doses of anthracyclines increase the risk of congestive heart failure (Jensen, 2006; McGowan et al., 2017). Although it is well established that anthracyclines cause cardiac damage, there is a gap in understanding mechanisms of cardiotoxicity and markers of susceptibility. Due to the clinical benefit of chemotherapy, it is now essential to find novel strategies to ameliorate side effects without compromising oncologic efficacy.

The Primary Prevention of Cardiovascular Disease with a Mediterranean Diet (PREDIMED) evaluated the effects of the Mediterranean diet on the prevention of cardiovascular disease (Estruch et al., 2018). The results of an interim analysis prompted early termination of the trial due to reductions in the cardiovascular event rates in the intervention groups. The PREDIMED study reported that consumption of the Mediterranean diet supplement with extra virgin olive oil (EVOO) and tree nuts lowered the risk of postmenopausal breast cancer by 51%. In another study assessing adherence to the Mediterranean diet and breast cancer risk, the Mediterranean diet was reported to reduce the risk of estrogen receptor‐negative breast cancer (Turati et al., 2018), suggesting Mediterranean diet consumption reduces TNBC breast cancer risk.

Due to the promising data on diet influencing the development of breast cancer and heart disease, we wanted to determine the impact of dietary patterns on chemotherapy‐induced cardiac dysfunction. We aimed to concurrently explore different dietary patterns effects on primary TNBC tumor growth, the development of metastatic lesions, the response to chemotherapy, and the development of doxorubicin‐induced cardiac damage. To do so, we employed two different TNBC models (4T1 and MDA‐MB‐231) consuming four different diets (standard rodent control diet, Western diet, Mediterranean diet, and a Western + Fish oil diet). We show that consuming a high‐fat diet increases primary breast tumor growth. However, the Mediterranean diet reduces lung metastatic burden and prevents the development of cardiac dysfunction.

## 2 MATERIAL AND METHODS

### 2.1 Compliance statements

The protocol was approved by the Animal Care and Use Committee of the Wake Forest School of Medicine (protocol #A18‐020), and all procedures were carried out in accordance with relevant guidelines and regulations.

### 2.2 Murine diet composition

All diets were formulated and purchased from Envigo (Indianapolis, IN). Standard rodent laboratory chow (control diet; TD.08806) contains approximately 10% kcal from fat and 9% by weight sucrose. We designed a 45% kcal from fat “Mediterranean” diet (TD.180299). The Mediterranean diet contains approximately 45% kcal from fat with olive oil being the main source, and 8% of fat is from fish oil. The Western diet (TD.180300) has reduced dietary fiber and increased sodium (7 g/kg). The Western diet contains approximately 45% kcal from fat with corn oil, palm oil, and milk‐fat being the main sources of dietary fat. The Western + fish oil diet (TD.180301) is designed to represent a Western diet background with 8% of the palm oil replaced by fish oil. See Table 1 for diet characteristics.

**TABLE 1:** Experimental diet composition and nutritional information

	Control diet (TD.08806)	Mediterranean diet (TD.180299)	Western diet (TD.180300)	Western diet + Fish oil supplementation (TD.180301)
Protein (% kcal)	20.5%	16.1%	15.9%	15.9%
Carbohydrates (% kcal)	69.1%	39%	39.6%	39.6%
Fat (% kcal)	10%	45%	44.5%	44.5%
Kcal/gram	3.6	4.4	4.4	4.4
Saturated fat	27%	18.3%	43.3%	41.5%
Monounsaturated fat	36.5%	67.3%	35.1%	33.9%
Polyunsaturated fat	36.5%	12.9%	20.5%	23%
*n*−6:*n*−3 ratio	7.1	3.0	34.4	6.2
Sucrose	11.2%	11.1%	27.4%	27.4%
Cholesterol (mg/kg)	60	136.4	246.5	337.3
Sodium (g/kg)	1.0	1.1	7	7
Potassium (g/kg)	3.6	8	3.9	3.9
Magnesium (mg/kg)	520	850	560	560
Calcium, g/k	5.6	5.5	5.5	5.5
Thiamin B1, ppm	7.3	5.3	5.3	5.3
Phosphorus, g/kg	3.5	3.1	3.1	3.1
Boric Acid, ppm	2.8	3.1	3.1	3.1
Zinc, ppm	42	45	45	45
Manganese, ppm	10.5	11.4	11.4	11.4
Vitamin K1, ppm	1.125	0.825	0.825	0.825
Fiber	3.7%	8%	8%	8%
Vitamin A (IU/kg)	6000	8400	6884	6884
Vitamin B_12_ (ug/kg)	3.75	11	11	11
Vitamin C (mg/kg)	0	500	0	0
Vitamin D (IU/kg)	1500	600	440	440
Vitamin E (IU/kg)	112.5	77.5	27.5	27.5
Folic Acid (mg/kg)	4.2	1.1	1.1	1.1

Note

Nutrient levels are calculated estimated and actual levels may vary from batch to batch.

### 2.3 Glucose tolerance test and body fat composition

Mice were overnight fasted with access to drinking water. Fasted blood glucose was measured using a OneTouch Ultra2 by LifeScan, Inc. (Milpitas, CA) with GenUltimate! Test Strips (Cat. No. 100‐10). Oral gavage of 2 g/kg sucrose was administered, and blood glucose was measured 15, 30, 60, and 120 min after gavage. Whole‐body fat composition was measured using EchoMRI^TM^ (Houston, TX).

### 2.4 *In vivo* TNBC tumor resection model

Female 3‐week old BALB/c mice were purchased from Charles River (Charleston, South Carolina) and placed on a control, Mediterranean, Western, or Western + Fish oil diet for 5 weeks. At 8‐weeks of age, mice were injected with 1 × 10^6^ 4T1‐luciferase breast cancer cells in the left inguinal fat pad. Primary tumor growth was monitored by IVIS and calipers weekly for 21 days. Primary tumors were then resected and IVIS imaging performed to confirm complete tumor removal. One week after tumor resection, mice received 3.3 mg/kg DOX I.V. for 3 weeks (cumulative dose of 9.9 mg/kg DOX), and the development of breast cancer lung metastases was monitored by weekly IVIS imaging. Transthoracic echocardiography was performed using a Vevo 2100 LAZR ultrasound system (FUJIFILM/VisualSonics, Inc.; Toronto, Canada) equipped with a 30 MHz linear array transducer. M‐mode short‐axis images were obtained to assess ejection fraction and fractional shortening. Mitral valve early filling velocities (E) and septal annular velocities (e’) were obtained using pulsed Doppler and tissue Doppler, respectively. E/e’ was calculated as an index of left ventricular (LV) filling pressure. To determine arterial stiffness the pulse wave velocity was calculated as follows: PWV = Distance (D)/Time (T), where D is the distance in mm between the ascending aorta site to descending aorta site and T = (R point of the EKG to the foot of the ascending aorta flow) − (R point of the EKG to the foot of the descending aorta flow) in msec (Rahimi et al., 2020). Cardiac function was measured in matched animals at 7 weeks of age, 10 weeks of age (tumor‐bearing), and 14 weeks of age (1 week post last DOX injection). At 14 weeks of age, the study was terminated, and mouse lung weight and heart weight were recorded. Mice that developed significant weight loss, shortness of breath, or other humane endpoints were removed from the study.

### 2.5 *In vivo* TNBC intra‐caudal artery injection lung metastases model

Female 4‐week old athymic (nu/nu) mice were purchased from Charles River (Charleston, South Carolina) and placed on a control, Mediterranean, Western, or a Western + Fish oil diet for 4 weeks. At 8‐weeks of age, mice were injected with 2.5 × 10^5^ MDA‐MB‐231‐luciferase breast cancer cells into the tail caudal artery. Mice were then injected with saline or 3.3 mg/kg DOX I.V. for 3 weeks. At 12 weeks of age, the study was terminated, and the lung metastases were visualized by fluorescent imaging. Lung weight, heart weight, and tibia length were measured and recorded.

### 2.6 RT‐PCR

RNA was isolated from snap‐frozen heart tissue using the Trizol reagent according to the manufacturer's protocol (Thermo Fisher Scientific). RNA concentration and integrity were measured with an Agilent 2100 Bioanalyzer with an RNA 6000 Nano LabChip (Agilent Technologies, Palo Alto, CA). cDNA was synthesized from 5 μg of total RNA using Superscript first strand RT‐PCR reagents as described by the manufacturer. RT‐PCR was then performed using SYBR (R) Green assay with specific primers for COL1A. HPRT served as an internal control. Results were quantified as cycle threshold (Ct) values and expressed as the ratio of target/control (Relative Gene Expression COL1A to HPRT) using the 2^−ΔΔCt^ method.

### 2.7 Immunohistochemistry

Hematoxylin and eosin (H&E) stained lung tissue was used to identify and quantify lung metastatic lesions. Tumor sections were stained against a Ki67 antibody (1:100) and visualized by 3,3'‐Diaminobenzidine (DAB) to demonstrate dietary effects on primary tumor proliferation. Cross‐sectional paraffin‐embedded cardiac tissue was stained using a picrosirius‐red protocol. Tibiae were isolated from whole legs, fixed in 10% formalin, and decalcified. Paraffin‐embedded bones were sectioned at 5 µm and placed on slides. H&E and bone histomorphometry was measured to determine bone volume fraction (BV/TV) and trabecular number was analyzed with BioQuant Osteo software (Figure S1).

### 2.8 Statistical analysis

Data are presented as the mean ± standard deviation (SD). Mouse body weight, tumor size, fractional shortening, ejection fraction, E/Eʹ, and pulse wave velocity over time were analyzed using two‐way ANOVA followed by a Tukey's multiple comparison test. Body fat composition, glucose AUC, tumor weight, time to lung metastatic lesion detection, lung weight, lung metastatic lesion quantification & size, COL1A gene expression, and heart weight were analyzed using one‐way ANOVA followed by a Tukey's multiple comparison test. Statistical significance was set at *p* < 0.05.

## 3 RESULTS

### 3.1 Dietary effects on metabolic parameters in the BALB/c 4T1‐luciferase tumor resection model

**FIGURE 1:**
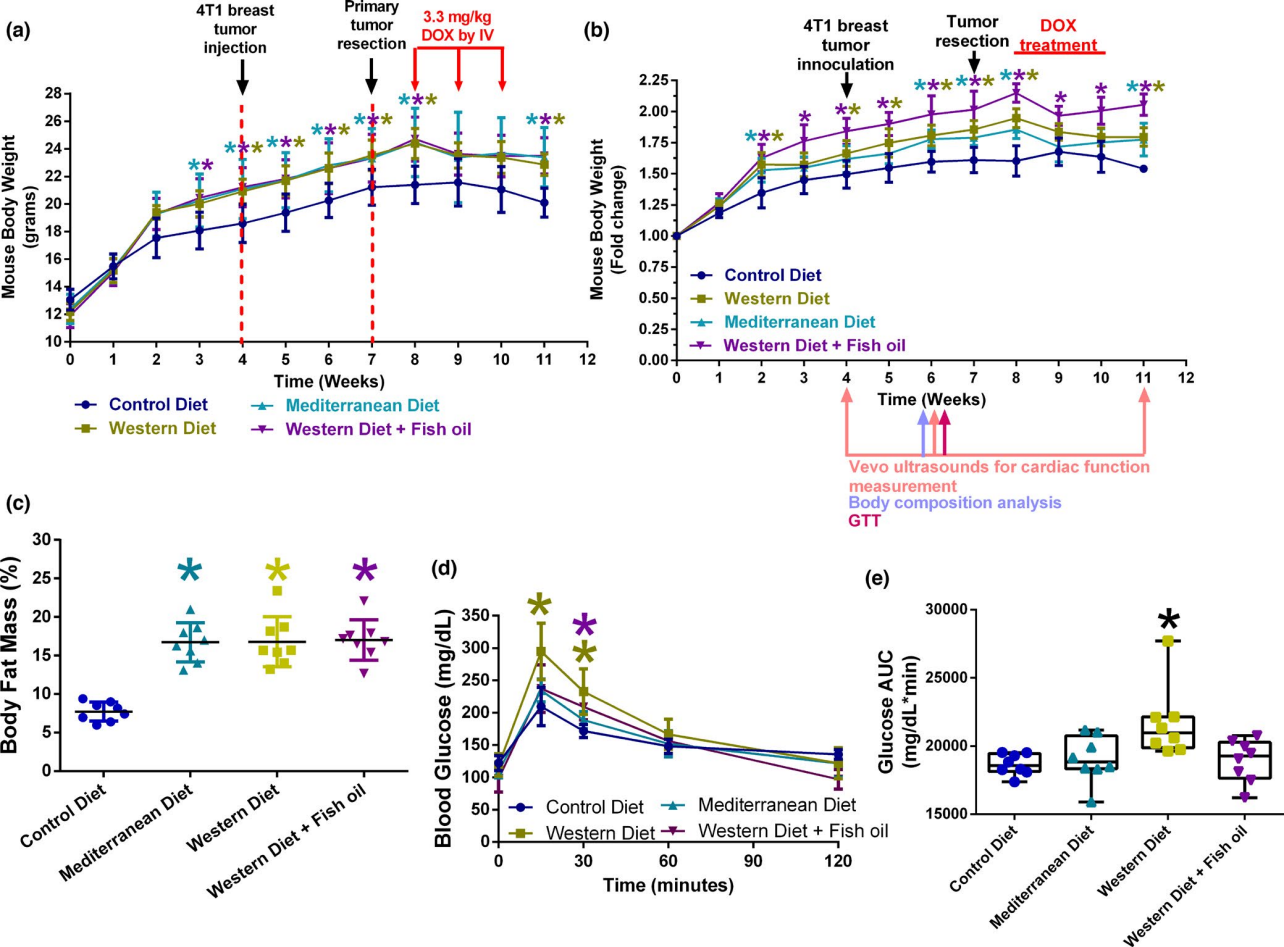
Dietary effects on metabolic parameters in the BALB/c 4T1‐luciferase tumor resection model. (a) Mouse body weight over the course of the study. *n* = 8–10 per diet. (b) Relative mouse body weight. Mouse body weight was normalized to baseline weight and graphed as a fold change of body weight over time. *n* = 8–10 per diet. (c) Body fat mass composition was determined by EchoMRI. *n* = 8 per diet. (d) Blood glucose levels overtime during a glucose challenge. *n* = 8 per diet. (e) Glucose area under the curve during a glucose challenge *n* = 8 per diet; *p < 0.05

Consumption of Mediterranean, Western, and Western + Fish oil diets increased body mass, composition, and glucose tolerance in comparison to mice‐fed the Control diet. Mice consuming the intervention diets (Mediterranean, Western, and Western + Fish oil) all exhibited increased total body weight compared to Control diet‐fed mice (Figure 1a). This trend was still observable when normalized to fold change in body weight (Figure 1b). Western Diet + Fish oil diet consuming mice had the highest average increase in fold change (approximately 2‐fold increase from baseline) of body weight, Western diet and Mediterranean diet‐fed mice were similar to each other (approximately 1.8‐fold increase from baseline), and Control diet‐fed mice had the lowest average fold change in body weight (approximately 1.5 fold increase from baseline).

The body fat composition of BALB/c mice fed intervention or control diets were measured using EchoMRI (TM). All three intervention diets were found to increase body fat mass percent compared to the Control diet (Figure 1c). However, there was no statistical or noticeable difference in body fat mass percent between Mediterranean, Western, or Western + Fish oil diet. Mice glucose tolerance was assessed using an oral glucose challenge. Western diet‐fed mice were found to have statistically elevated blood glucose at 15 and 30 min (Figure 1d), as well as an overall increase in glucose area under the curve compared to Control diet‐fed mice (Figure 1e). Western Diet + Fish oil‐fed mice only exhibited an increased blood glucose level at 30 min but did not exhibit an overall increase in glucose area under the curve (Figure 1d–e).

### 3.2 A high‐fat diet promotes primary tumor growth

**FIGURE 2:**
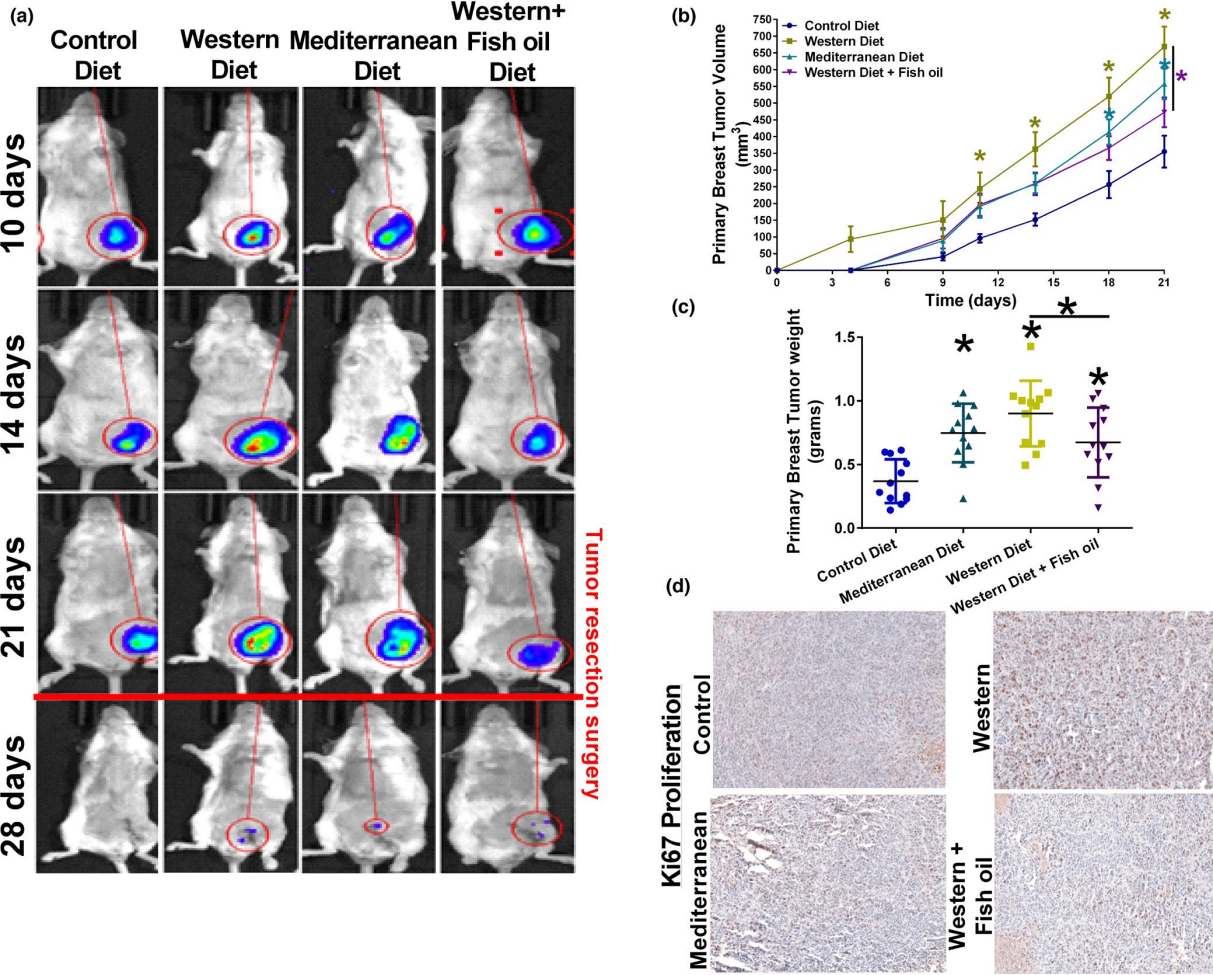
High fat diet promotes primary tumor growth regardless of the fat source. (a) Representative IVIS bioluminescent imaging of BALB/c mice bearing 4T1‐luciferase tumors on different diets over time. (b) Primary breast tumor volume measured every three days for 21 days. *n* = 12 per diet. (c) Tumor weight at 21 days after primary tumor resection surgery. *n* = 12 per diet. (d) Representative images of primary 4T1‐luciferase tumor Ki67 immunoreactivity as a marker of proliferation. *p < 0.05

The formation of 4T1‐luciferase orthotopic tumors in the mouse mammary fat pad was confirmed using IVIS imaging (Figure 2a). Tumors from mice fed high‐fat diets (Western, Western + Fish oil, and Mediterranean) all displayed increased tumor size compared to tumors from Control diet‐fed mice (Figure 2b). The tumors of Western diet‐fed mice were the first to have a measurable volume and exhibited the fastest growth, with a statistically significant increase in size eleven days post‐injection. The addition of fish oil to Western diet led to a decrease in tumor growth rate compared to the standard Western diet.

Tumor weight was measured at the time of resection. The tumors of high‐fat diet‐fed mice were all significantly larger than the tumors of Control diet‐fed mice (Figure 2c). Tumors of Western diet‐fed mice were the largest of all four diets. The addition of Fish oil to the Western diet significantly reduced tumor weight (Figure 2c). Tumor size correlated with cell proliferation as measured by Ki67 staining (Figure 2d). Tumors from Western diet‐fed mice contained increased Ki67 positive cells, and cell proliferation was reduced by supplementing the Western diet with fish oil.

### 3.3 Diet modulates the development of lung metastases and DOX responsiveness

**FIGURE 3:**
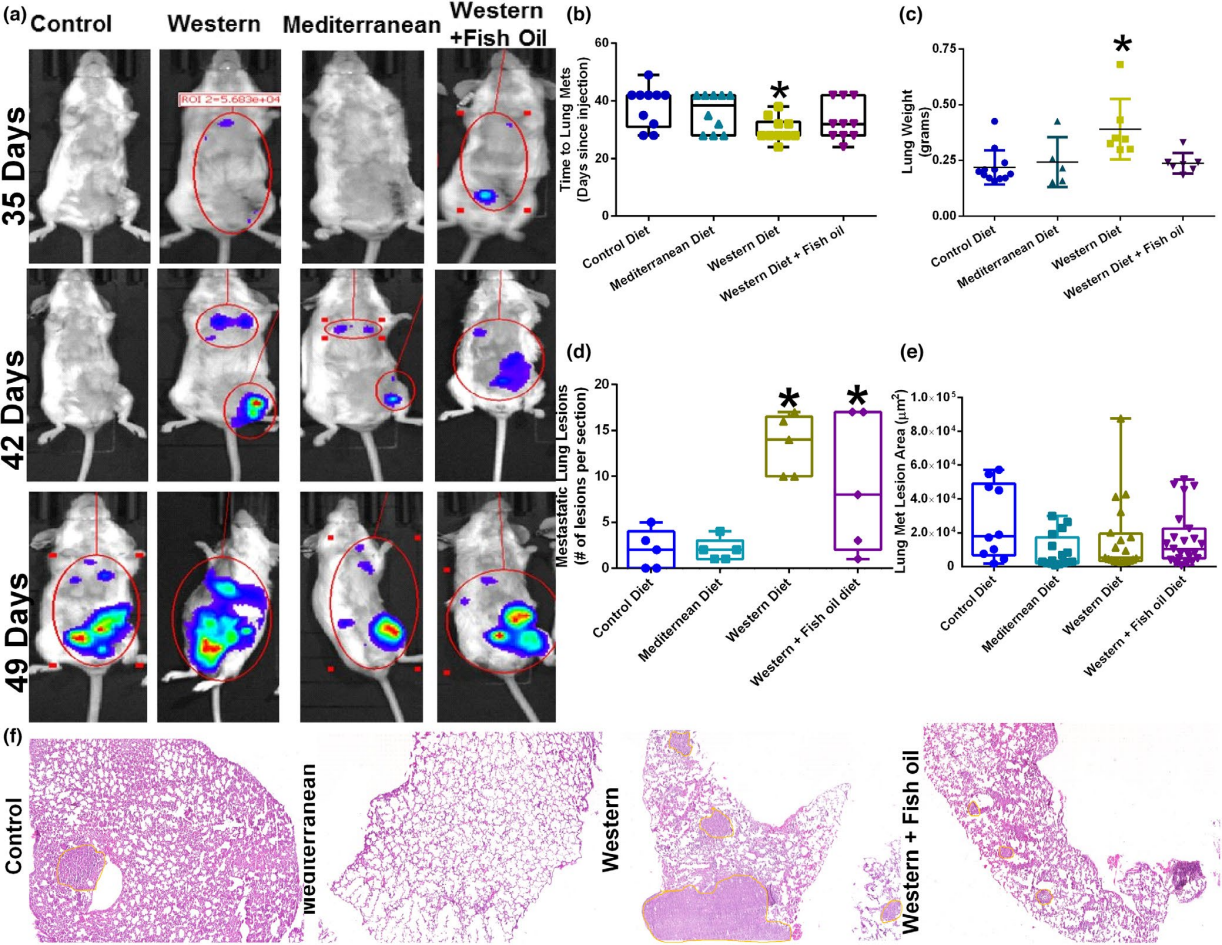
Diet differentially modulates the development of lung metastases and DOX responsiveness. (a) Representative IVIS bioluminescent imaging of BALB/c mice after 4T1‐luciferase tumor section surgery on different diets over time, indicating the development of lung metastases. (b) Time to lung metastatic lesion detection as determined by weekly IVIS in BALB/c mice fed differing dietary patterns. *n* = 10 per diet. (c) Lung weight at the sacrifice of mice that reached an endpoint. *n* = 5–12 per diet. (d) Lung metastatic lesions in paraffin‐embedded tissue were identified by H&E and counted as a number of lesions per section *n* = 5. (e) Average lung metastatic lesion area was determined by measuring the length and width of detected lung lesions of H&E stained tissue using ImageJ software. *n* = 5. (f) Representative H&E images of lung tissue from mice on each diet. Lung metastatic lesions are outlined in yellow. *p < 0.05

The development of lung metastases in post‐resection mice was confirmed via IVIS imaging (Figure 3a). Mice fed the Western diet were the fastest to develop lung metastases (Figure 3b). At the termination of the study, lung weight was recorded. Mice fed the Western diet had greatly increased lung weight compared to mice fed all other diets (Figure 3c). Both Mediterranean diet and Western + Fish oil diet‐fed mice had a discernible increase in lung weight, but they were not significantly larger than Control diet‐fed mice, nor significantly smaller than Western diet‐fed mice. Lung metastatic lesions were identified and quantified by H&E staining of paraffin‐embedded tissue (Figure 3d–f). Mice consuming a Western or Western + Fish oil diet displayed an increased number of lung metastatic lesions per section when compared with Control diet‐fed animals (Figure 3d). There was no significant difference in the average lung metastatic lesion area (Figure 3e).

### 3.4 Dietary patterns affect the development of DOX‐induced cardiac dysfunction

**FIGURE 4:**
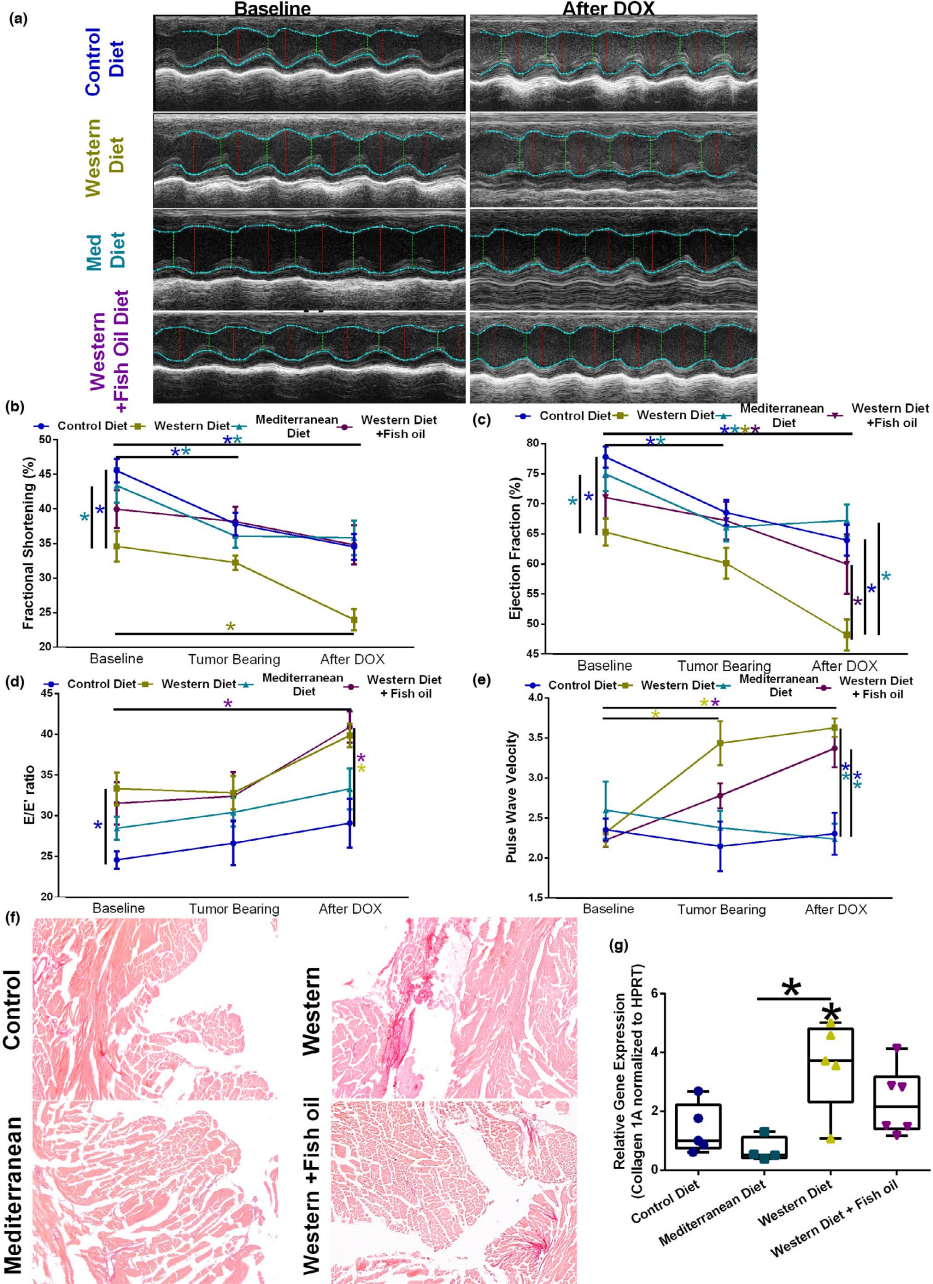
Dietary patterns affect the development of DOX‐induced cardiac dysfunction. (a) Representative images of M‐mode tracing using Vevo LAZR ultrasound in mice consuming different diets at baseline and after doxorubicin (DOX) therapy. (b) Fractional shortening (%) in mice consuming different diets at baseline, bearing primary 4T1‐luciferase breast tumor, and after DOX. *n* = 6. (c) Ejection fraction (%) in mice consuming different diets at baseline, bearing primary 4T1‐luciferase breast tumor, and after DOX. *n* = 6. (d) E/E' ratio in mice consuming different diets at baseline, bearing primary 4T1‐luciferase breast tumor, and after DOX. *n* = 6. (e) Pulse wave velocity in mice consuming different diets at baseline, bearing primary 4T1‐luciferase breast tumor, and after DOX. *n* = 6–9. (f) Representative images of picrosirius red staining in cardiac tissue from mice on each diet at the end of the study indicating collagen deposition. (g) Collagen 1A (*Col1a1*) gene expression in cardiac tissue from mice on each diet. *n* = 5. *p < 0.05

The cardiac function of TNBC‐resection model mice was measured using Vevo small animal ultrasound equipment (Figure 4a). Mice fed the Western diet exhibited a significant decrease in left ventricular systolic function, as measured by percent fractional shortening, compared to Control and Mediterranean diet‐fed mice (Figure 4b, “Baseline”). Supplementing the Western diet with fish oil created an observable, but non‐significant, increase in fractional shortening compared to the standard Western diet. Tumor burden significantly decreased percent fractional shortening in both Control and Mediterranean diet‐fed mice but did not further decrease fractional shortening in Western or Western + Fish oil diet‐fed mice (Figure 4b, “Tumor Bearing”). Control, Western, or Mediterranean diet‐fed mice all had decreased fractional shortening post‐DOX treatment compared to baseline. DOX treatment further decreased fractional shortening compared to tumor‐bearing time point in Western diet‐fed mice, though the decrease was not significant. Mediterranean diet‐fed mice did not exhibit any decrease in fractional shortening when tumor‐bearing or after DOX treatment compared to baseline. The percent change in fractional shortening from baseline to endpoint was significantly worse in Western diet‐fed animals (−31.6% compared with −20.6% in control diet‐fed mice; Table 2) which was not observed in Mediterranean or Western + fish oil diet‐fed mice.

**TABLE 2:** Cardiac function parameters in 4T1 murine study

	Control diet	Western diet	Mediterranean diet	Western + Fish oil diet
Fractional shortening				
Baseline	43.5 ± 2.5	36.7 ± 2.3	43.4 ± 6.2	40.0 ± 6.7
Endpoint	34.5 ± 4.2	22.8 ± 2.6	35.8 ± 6.1	34.8 ± 7.0
% Change in FS (Baseline to endpoint)	−20.6 ± 9.4	−31.6 ± 11.8	−17.0 ± 12.5	−10.3 ± 26.2
Ejection fraction				
Baseline	77.8 ± 3.9	65.3 ± 5.5	74.9 ± 7.0	71.0 ± 8.4
Endpoint	63.9 ± 5.7	48.2 ± 6.3	67.2 ± 6.5	60.0 ± 12.2
% Change in EF (Baseline to endpoint)	−14.2 ± 7.6	−29.8 ± 4.3	−10.1 ± 6.4	−15.3 ± 16.3
Heart rate (bpm)				
Baseline	432 ± 23	419 ± 27	395 ± 35	421 ± 43
Endpoint	444 ± 13	378 ± 41	371 ± 57	428 ± 45
LVPWd (mm)				
Baseline	0.72 ± 0.09	0.74 ± 0.06	0.71 ± 0.05	0.75 ± 0.04
Endpoint	0.73 ± 0.08	0.72 ± 0.04	0.74 ± 0.06	0.88 ± 0.10

Cardiac ejection fraction, a measurement of left ventricle function, was similarly affected by the intervention diets. Western diet‐fed mice had significantly reduced ejection fraction compared to Control diet‐fed mice (~12% less) and Mediterranean diet‐fed mice (~10% less) (Figure 4c “Baseline”). Western + Fish oil diet did not reduce baseline ejection fraction. Tumor burden significantly reduced ejection fraction in Control diet and Mediterranean diet‐fed mice (Figure 4c “Tumor Bearing”). After DOX treatment, mice on all diets had significantly reduced ejection fraction (Figure 4c “After DOX”). Western diet‐fed mice had significantly reduced ejection fraction compared to all other diets post DOX‐treatment. The percent change in ejection fraction from baseline to endpoint was significantly worse in Western diet‐fed animals (−29.8% compared with −14.2% in control diet‐fed mice; Table 2) which was not observed in Mediterranean or Western + fish oil diet‐fed mice.

Elevated E/E' ratio is considered a good predictor of heart failure. Mice consuming all three intervention diets had increased E/E' values at baseline, with Western diet‐fed mice having a significant increase in E/E' ratio (Figure 4d). E/E' values of tumor‐bearing mice were not significantly different from their relative baseline values. DOX treatment increased E/E' ratio in Western diet‐fed mice and with statistical significance in Western Diet + Fish oil‐fed mice. After DOX treatment, both Western diet and Western + Fish oil diet‐fed mice had a significantly higher E/E' ratio compared to Control diet‐fed mice. Mediterranean diet‐fed subjects were not significantly altered from Control diet‐fed mice.

Arterial stiffness, as measured by Pulse Wave Velocity, was not significantly altered by the intervention diets (Figure 4e “Baseline”). Neither tumor burden, nor DOX treatment, significantly altered the arterial stiffness of Control diet or Mediterranean diet‐fed mice. Mice fed Western diet exhibited a large, significant increase in arterial stiffness under tumor burden, which was reduced by supplementing fish oil (Figure 4e). After DOX treatment, Western diet‐fed mice, with or without fish oil, exhibited significantly increased arterial stiffness compared to Control diet and Mediterranean diet‐fed mice, as well as compared to their own baseline measurements.

Cardiac fibrosis is indicative of cardiac stress and damage. This was assessed using picrosirius red stain and collagen 1A gene expression. Mediterranean diet‐fed mice exhibited no increase in fibrosis via microscopy (Figure 4f). Western diet fed mice exhibited a stark increase in cardiac fibrosis. Fibrosis was ameliorated by supplementation with fish oil, though not to the extent that Western + Fish oil diet were comparable to Control diet‐fed mice (Figure 4f). These findings were supported by gene expression. Mediterranean diet‐fed mice exhibited Collagen 1A expression similar to Control diet‐fed mice. The cardiac tissue of Western diet‐fed mice expressed Collagen 1A significantly higher than Control diet or Mediterranean diet‐fed mice (Figure 4g).

### 3.5 High‐fat diet reduces the efficacy of DOX treatment on lung metastases

**FIGURE 5:**
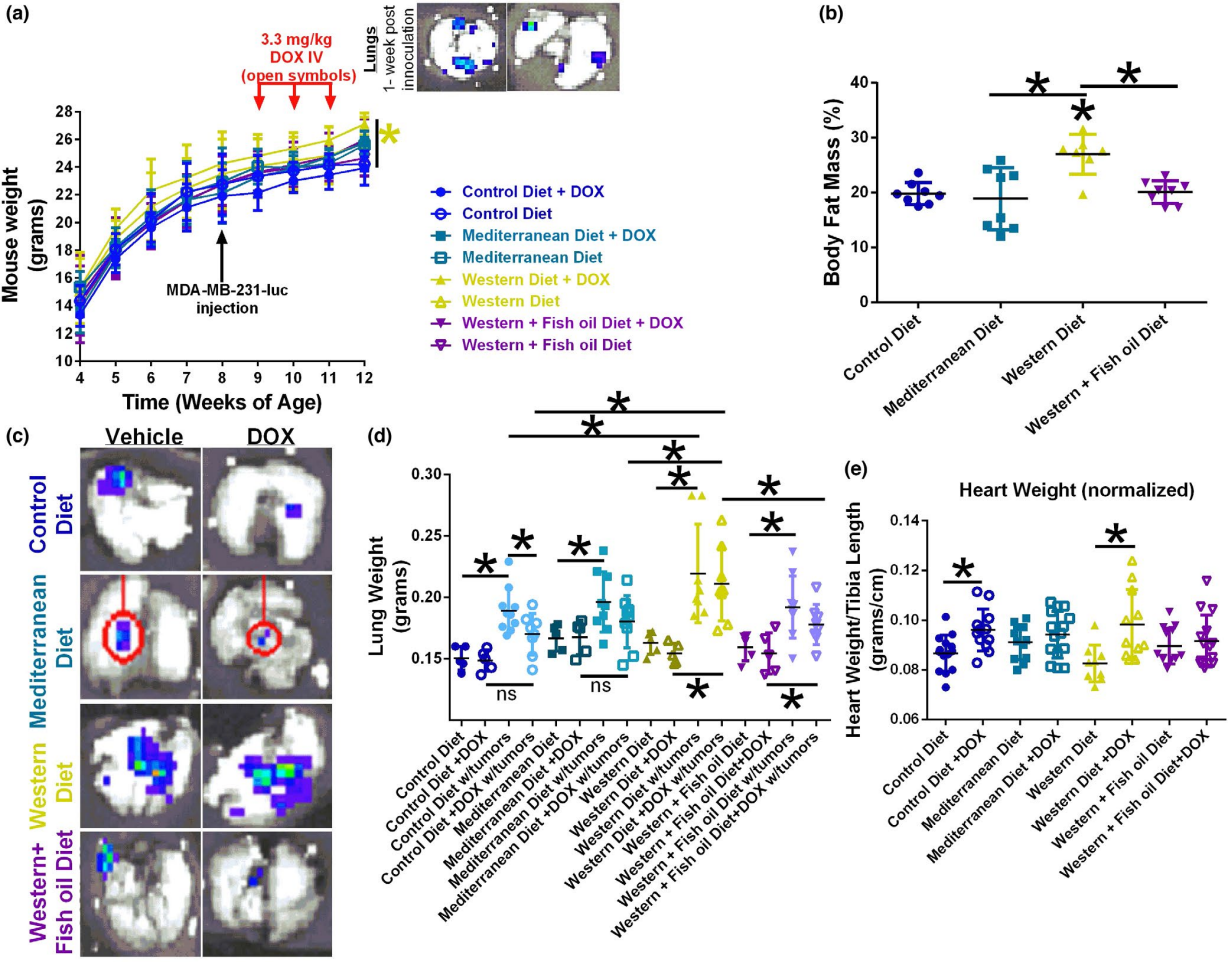
Lung MDA‐MB‐231 metastases tail vein injection model. (a) Mouse weight over time. Open symbols are animals treated with doxorubicin (DOX). (b) Body fat mass composition as determined by EchoMRI at 8 weeks of age before TNBC cell injection. *n* = 8. (c) Representative image of lung metastatic lesion formation in excised lungs from vehicle or DOX treated animals from each diet by IVIS. (d) Lung weight in non‐tumor‐bearing and tumor‐bearing animals treated with DOX. *n* = 5–9. (e) Heart weight (normalized to tibia length) in tumor‐free and tumor‐bearing mice on each diet treated with saline or DOX. *n* = 10–15. *p < 0.05

The weight of mice was recorded prior to (dietary effects) and post‐MDA‐MB‐231‐Luciferase injection (dietary and tumor burden effects). Western diet‐fed mice, without DOX treatment, were the only group to exhibit a statistical increase in weight compared to Control diet fed mice that were not treated with DOX (Figure 5a). The metastatic success of the intra‐caudal artery injected MDA‐MB‐231‐Luciferase cells was verified one‐week post‐injection (inset panel Figure 5a).

As seen in the 4T1‐Luciferase model (Figure 1a–b), Western diet‐fed mice had increased weight due to an increase in percent body fat mass (Figure 5b). Western diet‐fed mice exhibited a statistically increased body fat percent, by approximately 8%, compared to all other diets.

At the time of sacrifice, the tumors in Western diet‐fed mouse lungs were found to contain significantly larger metastatic lesions than all other diets (Figure 5c). IVIS visualization also revealed that DOX decreased metastatic lesion size in Control diet, Mediterranean diet, and Western + Fish oil diet‐fed mice, but not in Western diet‐fed mice (Figure 5c). Visualized results were confirmed by lung weight. Lung weight was significantly increased in all tumor‐bearing mice, regardless of diet (Figure 5d). Diet and DOX treatment did not have a discernible effect on lung weight in tumor‐free lungs. Tumors significantly increased the mass of lungs from mice fed all diets. Western diet‐fed mouse lungs containing tumors were significantly heavier than the tumor‐bearing lungs of mice fed Control diet, matching observations from IVIS imaging.

DOX treatment was effective in reducing tumor burden, as measured by lung weight, in Control diet and Mediterranean diet‐fed mice (Figure 5d). Post‐DOX treatment, lungs from mice fed Control diet and Mediterranean diet were not significantly different in weight from their respective DOX‐treated lungs without tumors. Lungs from mice fed Western diet or Western + Fish oil diet were still significantly heavier post‐DOX treatment compared to their respective tumor‐free DOX treated samples.

The cardiac effects of DOX treatment were assessed by measuring heart weight, which was then normalized to tibia length to account for differences in body size. DOX treatment significantly increased the heart weight/tibia length ratio in Control diet‐fed mice, and to a greater extent, in Western diet‐fed animals. This suggests western diet elevated DOX‐induced cardiac hypertrophy. These changes were not found in mice fed Mediterranean nor Western + Fish oil diets (Figure 5e).

## 4 DISCUSSION

Our findings demonstrate the impact of diet on the progression of TNBC; the ability of diet to modulate chemotherapy anti‐tumor efficacy; and the impact of diet on the development of chemotherapy dose‐limiting cardiac toxicities. This study demonstrates that not only is the level of fat content in a diet significant, but that the type of fat in a diet impacts these parameters as well. Elevated fat consumption increased body weight and body fat percent in both models used in this study. Additionally, increased weight was associated with a poor blood glucose regulation, as often observed in humans, which further compounded the deleterious effects of a high‐fat diet. In all cases, the high‐fat Western diet had the greatest impact on overall health, be it body fat percent, glucose tolerance, or cardiac function. These models allow us to simultaneously investigate the effects of diet, obesity, and associated co‐morbidities on breast cancer survival. The role of obesity on relapse‐free survival, and overall survival, is unclear. Some studies suggest obesity does not affect relapse‐free survival or overall survival of TNBC patients (Mei et al., 2018), though not all studies agree (Liu et al., 2018). However, there appears to be a consensus that obesity reduces therapeutic response in TNBC (Bonsang‐Kitzis et al., 2015; Naik et al., 2019), as was found in our preclinical study. These factors, combined with the increased risk of cardiac damage in obese patients with breast cancer (Kosalka et al., 2019), make this model, and protecting cardiac tissue from therapeutic damage, all the more vital to understand.

The type of fat consumed in a high‐fat diet differentially affected the two mouse models used in this study. BALB/c mice appear to be more sensitive to high fat consumption when compared with athymic nu/nu nude mice. All three intervention diets increased BALB/c body fat percent and body weight, while athymic nu/nu nude mice appear only susceptible to increased body fat via Western diet. Neither Mediterranean diet nor Western diet supplemented with fish oil led to an increase in body fat percent in nu/nu nude mice. These findings follow the well‐documented importance of genetic background and adipose‐immune‐inflammation interactions as regulators of an individual's response to a high‐fat diet (Francisco et al., 2018; Montgomery et al., 2013).

Consumption of a high‐fat diet increased the growth rate and size of primary tumors in the 4T1 BALB/c model, regardless of dietary pattern. This matches trends found in other 4T1‐high fat diet studies (Evangelista et al., 2019; Kim et al., 2011); though it is notable the source of fat calories varies between these reports. Western diet was notably the most detrimental, leading to the highest rate of tumor growth and the largest tumor size. Western diet‐fed mice were the first to develop lung metastases and were the only mice to have an increased lung weight. It is unclear whether this is due to diet‐induced metastases growth, inhibition of DOX treatment efficacy, or a combination of both. Similar results were found in the MDA‐MB‐231 metastasis nu/nu mouse model, where Western diet‐fed mice developed the largest tumors and had the highest lung weight. However, in this nu/nu model, both Mediterranean diet and supplementing Western diet with fish oil reduced lung metastasis compared to the 4T1 BALB/c model where only Mediterranean diet reduced lung metastatic lesion number. This may be due to differences in the mouse model immunogenicity and/or the primary TNBC cell line.

Diet was a significant factor in the efficacy of DOX treatment. In the MDA‐MB‐231 nu/nu nude mouse model, DOX treatment prevented growth or reduced lung metastases size in Control diet and Mediterranean diet‐fed mice so effectively that tumor‐bearing lung weight was the same as no‐tumor lung weight. This was not true of Western diet or Western + Fish oil diet‐fed mice, who still had a significant increase in lung mass post‐DOX treatment. This suggests Western diet reduces DOX efficacy. However, addition of fish oil to the Western diet background did improved DOX response compared to Western diet‐fed animal responses. Similar conclusions can be drawn from the 4T1 BALB/c model, though not as definitively. It is difficult to determine whether the differences in lung mass are a result of diet affecting DOX efficacy, metastatic lesion proliferation/apoptosis, or a combination of both.

Finally, diet was a significant regulator of cardiac health alone and cardiac damage mediated by DOX administration. Western diet and Western + Fish oil diet resulted in reduced cardiac diastolic function. Supplementing fish oil appears to be partially protective to the heart, in regards to systolic function preservation. These findings are consistent with some of the published data (reviewed in Serini et al., 2017). It is noteworthy that these studies are not ideal comparisons, as they utilize rats and sheep and different methodology than this study. Some studies showed PUFA supplementation showed no effect or increases anthracycline‐induced cardiac damage (Carbone et al., 2012; Matsui et al., 2002). However, other studies found PUFA supplementation to be protective of anthracycline‐induced cardiac damage (Schjott et al., 1996; Teng et al., 2010; Yu et al., 2013). PUFA and anthracycline dosage level and methodology appear to be significant factors in whether PUFA supplementation is protective or harmful. The study that indicates harmful effects was performed in sheep where the placebo was olive oil (high in monounsaturated fatty acids, previously shown to have health benefits) and a lower cumulative dose of DOX (3.6 mg/kg), which may confound data outcome interpretation (Carbone et al., 2012). While fish oil supplementation was found to be protective in this current study, further investigation of the cardio‐protective capabilities of PUFA is required with careful consideration of dose (sub‐optimal doses would not result in bioavailability and supra‐pharmacological doses would not be achievable in clinical population) and timing (administration of PUFA before or after DOX exposure may lead to differential results). Consumption of a Mediterranean diet improved both diastolic and systolic cardiac function when compared with Western diet‐fed subjects in response to chemotherapy, suggesting further dietary benefits of olive oil consumption or reduction in sugar intake in modulating chemotherapy‐mediated cardiotoxicities.

Breast cancer patients often experience accelerated bone loss as a side effect of systemic anti‐cancer therapies (Ramaswamy & Shapiro, 2003). DOX was reported to induce bone damage potentially leading to increased bone fracture risk (Fan et al., 2017). We show that consumption of a Western diet in combination with DOX administration led to a significant reduction in bone fraction (Figure S1) that was not observed in mice consuming Mediterranean diet, suggesting that diet may also play a key role in DOX‐mediated bone damage as another off‐target side effect induced by systemic chemotherapy.

These studies give general insight into the consequences of a Western diet on breast cancer progression, poor treatment efficacy, and cardiac damage. Supplementation with fish oil, and thus decrease in the *n*‐6:*n*‐3 ratio, was partially protective in some areas but did not fully prevent the aforementioned effects. Consumption of a Mediterranean diet while undergoing chemotherapy was more efficacious in reducing TNBC lung metastatic burden while simultaneously improving cardiac function when compared to DOX‐treated Western diet‐consuming subjects. Furthermore, understanding how diet affects cancer development and therapeutic outcomes is important in the age of individualized medicine. Given the societal and fiscal constraints on diet modification, these alternative strategies may be more deployable or easier to be made available.

## 5 STUDY LIMITATIONS

There are always study limitations that influence the outcomes and translational ability of the research. Our preclinical model uses young female BALB/c mice. While TNBC predominately affects younger women, this study may not accurately represent dietary influences in postmenopausal TNBC patients. Furthermore, it is well established that the BALB/c strain is resistant to diet‐induced obesity, which is observed in our study (animals on high‐fat diets do not surpass the 30% body fat composition that defines obesity). Therefore, our study portrays the effects of diets (independent of obesity) on TNBC growth, metastatic potential, DOX‐efficacy, and cardiac dysfunction. It is also important to note potential causal and temporal confounders of this study, mice bearing‐larger lung metastases would display impaired lung volume and have a greater impact on cardiac function. Therefore, the reduction in ejection fraction observed in Western diet consuming animal may represent indirect effects mediated by lung capacity. However, this is a translational relevant model as stage IV metastatic breast cancer patients may also develop cardiac toxicities.

## 6 CLINICAL PERSPECTIVE

The findings in this study agree with the general consensus of the field that diets high in salt, sugar, and saturated fats increase tumor growth and metastases, while concurrently decreasing DOX anti‐tumor efficacy. Few studies investigate dietary effects on both tumor and heart simultaneously. We now show in a tumor‐bearing model how diet can affect cancer therapy sequelae, such as chemotherapy‐induced cardiac damage. In light of these findings, it is important that the general public be made aware of the influence of diet on health and that breast cancer patients are made to understand the importance of diet regulation during treatment.

## CONFLICT OF INTEREST

Authors report that they have no conflict of interest.

## AUTHOR CONTRIBUTIONS

Manuel U. Ramirez, Kenysha YJ Clear, Zipporah Cornelius, Alaa Bawaneh, Yismeilin R. Feliz‐Mosquea, Adam S. Wilson, Alistaire D. Ruggiero, Nildris Cruz‐Diaz, Lihong Shi, and Bethany A. Kerr performed experiments and obtained the data included in this manuscript. Nildris Cruz‐Diaz, Bethany A. Kerr, David R. Soto‐Pantoja, and Katherine L. Cook analyzed the data. Manuel U. Ramirez, David R. Soto‐Pantoja, and Katherine L. Cook wrote the manuscript. Katherine L. Cook contributed to the conceptual design of experiments. David R. Soto‐Pantoja, Bethany A. Kerr, and Katherine L. Cook supervised the study. All authors discussed the results and commented on the manuscript.

## Supporting information



Supplementary Material

## References

[phy215192-bib-0001] Bonsang‐Kitzis, H. , Chaltier, L. , Belin, L. , Savignoni, A. , Rouzier, R. , Sablin, M.‐P. , Lerebours, F. , Bidard, F.‐C. , Cottu, P. , Sastre‐Garau, X. , Laé, M. , Pierga, J.‐Y. , & Reyal, F. (2015). Beyond axillary lymph node metastasis, BMI and menopausal status are prognostic determinants for triple‐negative breast cancer treated by neoadjuvant chemotherapy. PLoS One, 10, e0144359. 10.1371/journal.pone.0144359 26684197 PMC4686172

[phy215192-bib-0002] Bradshaw, P. T. , Stevens, J. , Khankari, N. , Teitelbaum, S. L. , Neugut, A. I. , & Gammon, M. D. (2016). Cardiovascular disease mortality among breast cancer survivors. Epidemiology, 27, 6–13. 10.1097/EDE.0000000000000394 26414938 PMC4666721

[phy215192-bib-0003] Carbone, A. , Psaltis, P. J. , Nelson, A. J. , Metcalf, R. , Richardson, J. D. , Weightman, M. , Thomas, A. , Finnie, J. W. , Young, G. D. , & Worthley, S. G. (2012). Dietary omega‐3 supplementation exacerbates left ventricular dysfunction in an ovine model of anthracycline‐induced cardiotoxicity. Journal of Cardiac Failure, 18, 502–511. 10.1016/j.cardfail.2012.03.005 22633309

[phy215192-bib-0004] Estruch, R. , Ros, E. , Salas‐Salvado, J. , Covas, M.‐I. , Corella, D. , Arós, F. , Gómez‐Gracia, E. , Ruiz‐Gutiérrez, V. , Fiol, M. , Lapetra, J. , Lamuela‐Raventos, R. M. , Serra‐Majem, L. , Pintó, X. , Basora, J. , Muñoz, M. A. , Sorlí, J. V. , Martínez, J. A. , Fitó, M. , Gea, A. , … Martínez‐González, M. A. (2018). Primary prevention of cardiovascular disease with a mediterranean diet supplemented with extra‐virgin olive oil or nuts. New England Journal of Medicine, 378, e34. 10.1056/NEJMoa1800389 29897866

[phy215192-bib-0005] Evangelista, G. C. M. , Salvador, P. A. , Soares, S. M. A. , Barros, L. R. C. , Xavier, F. H. D. C. , Abdo, L. M. , Gualberto, A. C. M. , Macedo, G. C. , Clavijo‐Salomon, M. A. , & Gameiro, J. (2019). 4T1 mammary carcinoma colonization of metastatic niches is accelerated by obesity. Frontiers in Oncology, 9, 685. 10.3389/fonc.2019.00685 31616626 PMC6764084

[phy215192-bib-0006] Fan, C. , Georgiou, K. R. , Morris, H. A. , McKinnon, R. A. , Keefe, D. M. K. , Howe, P. R. , & Xian, C. J. (2017). Combination breast cancer chemotherapy with doxorubicin and cyclophosphamide damages bone and bone marrow in a female rat model. Breast Cancer Research and Treatment, 165, 41–51. 10.1007/s10549-017-4308-3 28550626

[phy215192-bib-0007] Foulkes, W. D. , Smith, I. E. , & Reis‐Filho, J. S. (2010). Triple‐negative breast cancer. New England Journal of Medicine, 363, 1938–1948. 10.1056/NEJMra1001389 21067385

[phy215192-bib-0008] Francisco, V. , Pino, J. , Campos‐Cabaleiro, V. , Ruiz‐Fernández, C. , Mera, A. , Gonzalez‐Gay, M. A. , Gómez, R. , & Gualillo, O. (2018). Obesity, fat mass and immune system: Role for leptin. Frontiers in Physiology, 9, 640. 10.3389/fphys.2018.00640 29910742 PMC5992476

[phy215192-bib-0009] Gernaat, S. A. M. , Ho, P. J. , Rijnberg, N. , Emaus, M. J. , Baak, L. M. , Hartman, M. , Grobbee, D. E. , & Verkooijen, H. M. (2017). Risk of death from cardiovascular disease following breast cancer: A systematic review. Breast Cancer Research and Treatment, 164, 537–555. 10.1007/s10549-017-4282-9 28503723 PMC5495872

[phy215192-bib-0010] Guenancia, C. , Lefebvre, A. , Cardinale, D. , Yu, A. F. , Ladoire, S. , Ghiringhelli, F. , Zeller, M. , Rochette, L. , Cottin, Y. , & Vergely, C. (2016). Obesity as a risk factor for anthracyclines and trastuzumab cardiotoxicity in breast cancer: A systematic review and meta‐analysis. Journal of Clinical Oncology, 34, 3157–3165. 10.1200/JCO.2016.67.4846 27458291 PMC5569689

[phy215192-bib-0011] Isakoff, S. J. (2010). Triple‐negative breast cancer: Role of specific chemotherapy agents. Cancer Journal, 16, 53–61. 10.1097/PPO.0b013e3181d24ff7 PMC2882502 20164691

[phy215192-bib-0012] Jensen, B. V. (2006). Cardiotoxic consequences of anthracycline‐containing therapy in patients with breast cancer. Seminars in Oncology, 33, S15–S21. 10.1053/j.seminoncol.2006.04.022 16781285

[phy215192-bib-0013] Kim, E. J. , Choi, M. R. , Park, H. , Kim, M. , Hong, J. E. , Lee, J.‐Y. , Chun, H. S. , Lee, K. W. , & Yoon Park, J. H. (2011). Dietary fat increases solid tumor growth and metastasis of 4T1 murine mammary carcinoma cells and mortality in obesity‐resistant BALB/c mice. Breast Cancer Research, 13, R78. 10.1186/bcr2927 21834963 PMC3236342

[phy215192-bib-0014] Kosalka, P. , Johnson, C. , Turek, M. , Sulpher, J. , Law, A. , Botros, J. , Dent, S. , & Aseyev, O. (2019). Effect of obesity, dyslipidemia, and diabetes on trastuzumab‐related cardiotoxicity in breast cancer. Current Oncology (Toronto, Ont.), 26, e314–e321. 10.3747/co.26.4823 PMC6588065 31285674

[phy215192-bib-0015] Liu, Y. L. , Saraf, A. , Catanese, B. , Lee, S. M. , Zhang, Y. , Connolly, E. P. , & Kalinsky, K. (2018). Obesity and survival in the neoadjuvant breast cancer setting: Role of tumor subtype in an ethnically diverse population. Breast Cancer Research and Treatment, 167, 277–288. 10.1007/s10549-017-4507-y 28948418 PMC5790631

[phy215192-bib-0016] Matsui, H. , Morishima, I. , Hayashi, K. , Kamiya, H. , Saburi, Y. , & Okumura, K. (2002). Dietary fish oil does not prevent doxorubicin‐induced cardiomyopathy in rats. Canadian Journal of Cardiology, 18, 279–286. 11907617

[phy215192-bib-0017] McGowan, J. V. , Chung, R. , Maulik, A. , Piotrowska, I. , Walker, J. M. , & Yellon, D. M. (2017). Anthracycline chemotherapy and cardiotoxicity. Cardiovascular Drugs and Therapy, 31, 63–75. 10.1007/s10557-016-6711-0 28185035 PMC5346598

[phy215192-bib-0018] Mei, L. , He, L. , Song, Y. , Lv, Y. , Zhang, L. , Hao, F. , & Xu, M. (2018). Association between obesity with disease‐free survival and overall survival in triple‐negative breast cancer: A meta‐analysis. Medicine (Baltimore), 97, e0719. 10.1097/MD.0000000000010719 29742734 PMC5959383

[phy215192-bib-0019] Montgomery, M. K. , Hallahan, N. L. , Brown, S. H. , Liu, M. , Mitchell, T. W. , Cooney, G. J. , & Turner, N. (2013). Mouse strain‐dependent variation in obesity and glucose homeostasis in response to high‐fat feeding. Diabetologia, 56, 1129–1139. 10.1007/s00125-013-2846-8 23423668

[phy215192-bib-0020] Naik, A. , Monjazeb, A. M. , & Decock, J. (2019). The obesity paradox in cancer, tumor immunology, and immunotherapy: Potential therapeutic implications in triple negative breast cancer. Frontiers in Immunology, 10, 1940. 10.3389/fimmu.2019.01940 31475003 PMC6703078

[phy215192-bib-0021] Patnaik, J. L. , Byers, T. , DiGuiseppi, C. , Dabelea, D. , & Denberg, T. D. (2011). Cardiovascular disease competes with breast cancer as the leading cause of death for older females diagnosed with breast cancer: A retrospective cohort study. Breast Cancer Research, 13, R64. 10.1186/bcr2901 21689398 PMC3218953

[phy215192-bib-0022] Patnaik, J. L. , Byers, T. , Diguiseppi, C. , Denberg, T. D. , & Dabelea, D. (2011). The influence of comorbidities on overall survival among older women diagnosed with breast cancer. Journal of the National Cancer Institute, 103, 1101–1111. 10.1093/jnci/djr188 21719777 PMC3139585

[phy215192-bib-0023] Perez, I. E. , Taveras Alam, S. , Hernandez, G. A. , & Sancassani, R. (2019). Cancer therapy‐related cardiac dysfunction: An overview for the clinician. Clinical Medicine Insights: Cardiology, 13, 117954681986644. 10.1177/1179546819866445 PMC6664629 31384135

[phy215192-bib-0024] Pierobon, M. , & Frankenfeld, C. L. (2013). Obesity as a risk factor for triple‐negative breast cancers: A systematic review and meta‐analysis. Breast Cancer Research and Treatment, 137, 307–314. 10.1007/s10549-012-2339-3 23179600

[phy215192-bib-0025] Protani, M. , Coory, M. , & Martin, J. H. (2010). Effect of obesity on survival of women with breast cancer: Systematic review and meta‐analysis. Breast Cancer Research and Treatment, 123, 627–635. 10.1007/s10549-010-0990-0 20571870

[phy215192-bib-0026] Rahimi, O. , Kirby, J. , Varagic, J. , Westwood, B. , Tallant, E. A. , & Gallagher, P. E. (2020). Angiotensin‐(1–7) reduces doxorubicin‐induced cardiac dysfunction in male and female Sprague‐Dawley rats through antioxidant mechanisms. American Journal of Physiology‐Heart and Circulatory Physiology, 318, H883–H894. 10.1152/ajpheart.00224.2019 32083974

[phy215192-bib-0027] Ramaswamy, B. , & Shapiro, C. L. (2003). Osteopenia and osteoporosis in women with breast cancer. Seminars in Oncology, 30, 763–775. 10.1053/j.seminoncol.2003.08.028 14663777

[phy215192-bib-0028] Schjott, J. , Brurok, H. , Jynge, P. , & Bjerve, K. S. (1996). Effects of eicosapentaenoic acid and docosahexaenoic acid diet supplement on tolerance to the cardiotoxicity of epirubicin and to ischaemia reperfusion in the isolated rat heart. Pharmacology and Toxicology, 79, 65–72. 10.1111/j.1600-0773.1996.tb00244.x 8878248

[phy215192-bib-0029] Serini, S. , Ottes Vasconcelos, R. , Nascimento Gomes, R. , & Calviello, G. (2017). Protective effects of omega‐3 PUFA in anthracycline‐induced cardiotoxicity: A critical review. International Journal of Molecular Sciences, 18, 2689. 10.3390/ijms18122689 PMC5751291 29231904

[phy215192-bib-0030] Siegel, R. L. , Miller, K. D. , Fuchs, H. E. , & Jemal, A. (2021). Cancer statistics, 2021. CA: A Cancer Journal for Clinicians, 71, 7–33. 10.3322/caac.21654 33433946

[phy215192-bib-0031] Sun, H. , Zou, J. , Chen, L. , Zu, X. , Wen, G. , & Zhong, J. (2017). Triple‐negative breast cancer and its association with obesity. Molecular and Clinical Oncology, 7, 935–942. 29285353 10.3892/mco.2017.1429 PMC5740844

[phy215192-bib-0032] Teng, L. L. , Shao, L. , Zhao, Y. T. , Yu, X. , Zhang, D. F. , & Zhang, H. (2010). The beneficial effect of n‐3 polyunsaturated fatty acids on doxorubicin‐induced chronic heart failure in rats. Journal of International Medical Research, 38, 940–948. 10.1177/147323001003800320 20819430

[phy215192-bib-0033] Turati, F. , Carioli, G. , Bravi, F. , Ferraroni, M. , Serraino, D. , Montella, M. , Giacosa, A. , Toffolutti, F. , Negri, E. , Levi, F. , & La Vecchia, C. (2018). Mediterranean diet and breast cancer risk. Nutrients, 10, 326. 10.3390/nu10030326 PMC5872744 29518016

[phy215192-bib-0034] Yu, X. , Cui, L. , Zhang, Z. , Zhao, Q. , & Li, S. (2013). Alpha‐Linolenic acid attenuates doxorubicin‐induced cardiotoxicity in rats through suppression of oxidative stress and apoptosis. Acta Biochimica Et Biophysica Sinica (Shanghai), 45, 817–826. 10.1093/abbs/gmt082 23896563

