# Supplementary material for: Diet impacts triple‐negative breast cancer growth, metastatic potential, chemotherapy responsiveness, and doxorubicin‐mediated cardiac dysfunction
Source: Physiol Rep. 2022 Apr 19;10(8):e15192. doi: 10.14814/phy2.15192 (PMC9017973; doi:10.14814/phy2.15192)

Supplemental Figure and Legend.

Supplemental Figure S1. Doxorubicin-induced bone loss during lung MDA-MB-231 metastases. **A**. Bone fraction (BV/TV; Bone volume :Total volume ratio) and **B.** Trabecular number were measured by bone histomorphometry using Bioquant Osteo Image Analysis Software in tumor-bearing mice on each diet treated with saline or DOX. n=3-5. *p<0.05 one-way ANOVA followed by a Tukey's multiple comparison test.

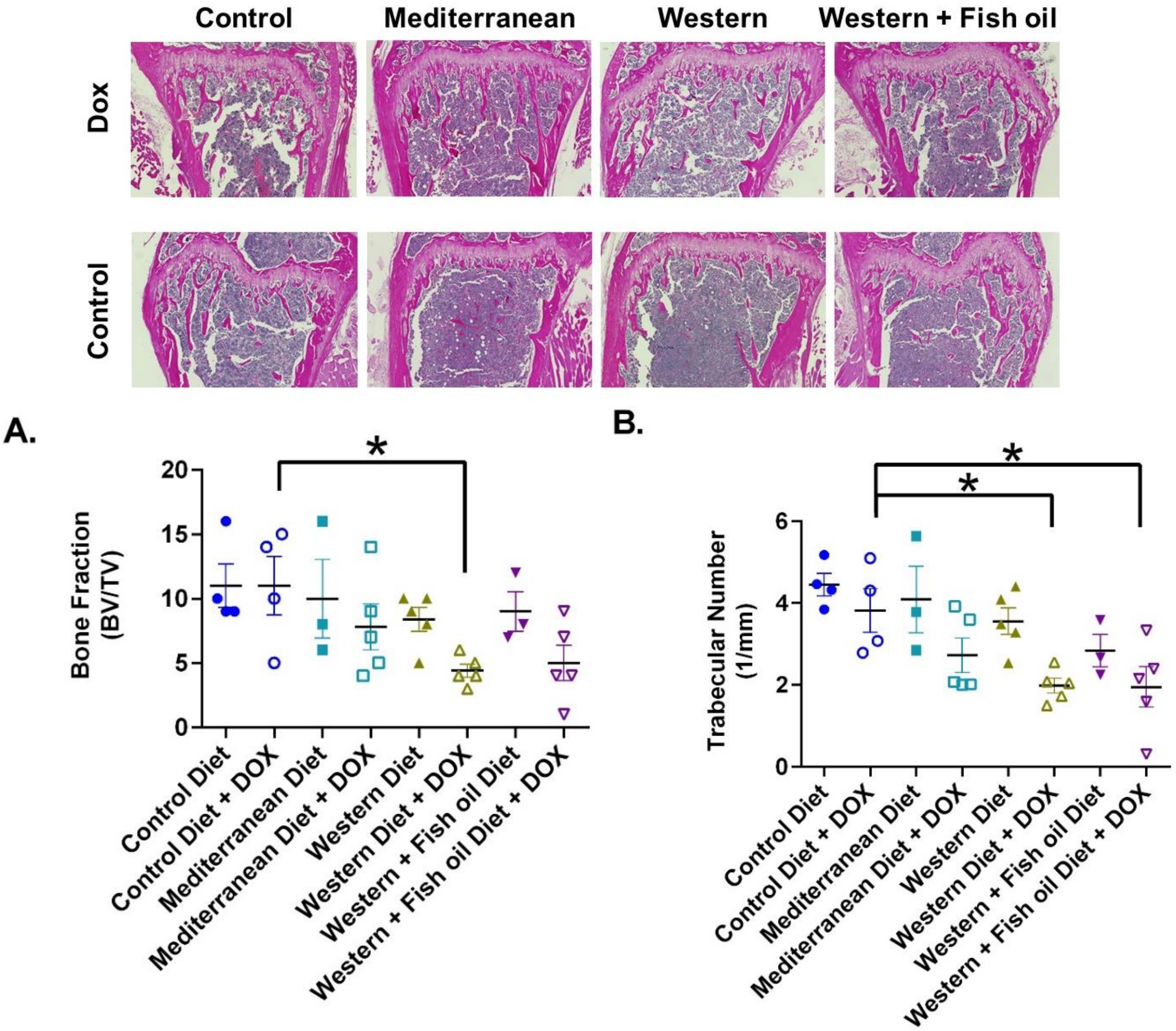

Supplement: Supplementary file 1 — Supplementary Material [file PHY2-10-e15192-s001.pdf]
